# CTL-Derived Exosomes Enhance the Activation of CTLs Stimulated by Low-Affinity Peptides

**DOI:** 10.3389/fimmu.2019.01274

**Published:** 2019-06-04

**Authors:** Shu-Wei Wu, Lei Li, Yan Wang, Zhengguo Xiao

**Affiliations:** ^1^Department of Animal and Avian Sciences, University of Maryland, College Park, MD, United States; ^2^Department of Cell Biology and Molecular Genetics, University of Maryland, College Park, MD, United States

**Keywords:** CTLs, IL-12, exosomes, activation, low-affinity, N4 peptides

## Abstract

Cytotoxic T cells (CTLs) bind to peptides presented by MHC I (pMHC) through T cell receptors of various affinities. Low-affinity CTLs are important for the control of intracellular pathogens and cancers; however, the mechanisms by which these lower affinity CTLs are activated and maintained are not well understood. We recently discovered that fully activated CTLs stimulated by strong-affinity peptides in the presence of IL-12 are able to secrete exosomes that, in turn, stimulate bystander CTLs without requiring the presence of antigen. We hypothesized that exosomes secreted by high-affinity CTLs could strengthen the activation of low-affinity CTLs. Naive OT-I CD8+ cells were stimulated with altered N4 peptides of different affinities in the presence or absence of Exo. The presence of Exo preferentially increased cell proliferation and enhanced the production of IFNγ in CTLs stimulated by low-affinity peptides. The expression of granzyme B (GZB) was augmented in all affinities, with higher GZB production in low-affinity stimulated CTLs than in high-affinity stimulated ones. Exosomes promoted the rapid activation of low-affinity CTLs, which remained responsive to exosomes for a prolonged duration. Unexpectedly, exosomes could be induced quickly (24 h) following CTL activation and at a higher quantity per cell than later (72 h). While exosome protein profiles vary significantly between early exosomes and their later-derived counterparts, both appear to have similar downstream functions. These results reveal a potential mechanism for fully activated CTLs in activating lower-affinity CTLs that may have important implications in boosting the function of low-affinity CTLs in immunotherapy for cancers and chronic viral infections.

## Introduction

Single positive CD4+ or CD8+ T cells mature in and migrate from the thymus following positive and negative selection to ensure this T cell pool remains self-restricted and non-autoaggressive (1, 2). Selection depends upon the affinity of the T cell receptor (TCR) for the peptide/MHC complex (pMHC) (2–7). Most CTLs (CD8+ T cells) are low-affinity (8, 9), but high-affinity CTLs are considered more essential to the immune response due to their more robust function and increased sensitivity to detection (10–12). The presence of CTLs with diverse affinities has been confirmed throughout the immune response (13, 14) via improved, more sensitive assays for detecting low-affinity CTLs (15–17). Of note, a similarly prominent existence of low-affinity polyclonal CD4+ T cell responses has also been reported (14, 18, 19). Low-affinity CTLs are important to fighting infection and malignant cells (12, 18, 20–22), particularly in the presence of multiple epitopes or where immune escape mutations occur (23). A greater breadth of recruited TCR affinities has been positively associated with improved host protection (12, 24). Low-affinity CTLs can become effectors despite the reduced magnitude of their immune activity compared to their high-affinity counterparts (25). Memory low-affinity CTLs are induced and maintained during infection (12, 26, 27) and can mount a robust recall response (28). How this low-affinity CTL response is initiated and maintained, however, is not well-understood (12, 27).

It has been elegantly demonstrated that affinity affects the kinetics of CTL expansion and contraction as well as egress from draining lymph nodes (12). Low affinity-primed CTLs expand to a lesser degree and contract earlier than high affinity-primed CTLs, and also exit lymphoid organs sooner and are released into circulation earlier in the adaptive immune response. These low affinity-primed CTLs may contribute to the early control of infection, whereas high affinity-primed CTLs are released later to take over the remainder of the CTL response (12). This was further supported by another recent report that presented evidence that low affinity-primed CTLs accumulate at efferent lymphatic vessels and are disseminated earlier than high affinity-primed ones, leading to rapid elimination of targets outside the lymph nodes (27). Low affinity-primed CTLs may be at least partially responsible for early control of microbial infections, serving as a critical part of the adaptive immune response together with their high-affinity counterparts (27).

TCR signaling may differ between low- and high-affinity CTLs. Reduced TCR affinity is generally associated with a reduced CTL response (12, 25, 27, 29–31). However, how the activation of CTLs is directly affected by TCR affinity remains controversial (5, 29, 30, 32–41). In a recent report, CTLs stimulated with peptides of different affinities nonetheless achieved a similar effector protein profile (42). The TCR signaling triggered by weak ligands may be different from that induced by strong ligands (43), as demonstrated by a unique pattern of ZAP-70 phosphorylation (44), representing an altered TCR activation pathway not explained by dose effects (44, 45). In addition, TCR affinity seems to affect transcription factor expression. Low affinity is associated with high eomes expression at high antigen doses (46), whereas strong TCR affinity reduces the ratio of Bcl6 to Blimp-1 and eomes to T-bet (46, 47). In addition, strong affinity induces higher expression, and low affinity induces reduced expression of both BATF and IRF4 (27). These reports suggest that low- and high-affinity CTLs may possess different TCR signaling pathways and may also be responding differently to other stimuli, such as exosomes.

Recently, we reported that antigen-stimulated CTLs secrete exosomes and that the presence of IL-12 changes their morphology and influences the enrichment of the proteins contained therein (48). More important, these IL-12-conditioned, CTL-derived exosomes can activate bystander naive CTLs without antigen stimulation (48). In this project, we examined the functions of these CTL-derived exosomes on CTLs stimulated with altered peptides of different affinities, using a simple OT-I cell *in vitro* stimulation model.

## Materials and Methods

### Purification of Naive OT-I CD8+ T Cells

OT-I mice were euthanized, and peripheral lymph nodes were collected. The harvested lymph nodes were homogenized in 15 mL glass grinders in Allos medium (49, 50). After washing with Allos medium several times and filtering through a 70 μm nylon filter (VWR, Radnor, PA), cells were incubated together with FITC-labeled antibodies specific to B220, CD4, CD44, CD11c, and I-Ab for negative selection (Biolegend, San Diego, CA). The suspension was subsequently incubated with Anti-FITC conjugated magnetic MicroBeads (Miltenyi Biotech, Auburn CA) and passed through separation columns attached to a MACS magnet. Cells that did not bind to the column were collected, and their purity was confirmed (>95% CD8+ and <0.5% CD44hi cells).

### Activation of Naive CTLs for Exosome Production

Flat-bottom Microtiter plates (Greiner bio-one, Frickenhausen, Germany) were coated with recombinant MHC I (DimerX H-2Kb: Ig fusion protein; BD Pharmingen, San Jose, CA) and the costimulatory molecule B7-1/Fc chimeric protein (R&D Systems, Minneapolis, MN) (49, 50). The coated plates were pulsed with N4 peptides. This MHC I/N4 plus B7-1 provided two signals (2SI): the first signal to the specific TCR expressed on the surface of OT-I CD8+ T cells, and the second signal (costimulation), thus designated as “2SI” stimulation. For 2SI stimulation, purified naive OT-I CD8+ T cells were placed at 3 × 10^5^ cells in 1.5 mL Allos medium in each well of a 24-well plate with IL-2 at 2.5 U/mL. For three signal stimulation (3SI), naive OT-I CD8 T cells were stimulated with 2SI and supplemented with 2 U/mL of murine rIL-12 (R&D Systems, Minneapolis, MN), as previously described (48, 51). Supernatant from 2SI or 3SI stimulated CTLs was harvested three days after stimulation for exosome purification. Exosomes from 2SI were designated as “2SI-exo,” whereas those from 3SI as “Exo” or “3SI-exo.” D1 exosomes (D1E) were purified from the CTL supernatant after a one-day stimulation with 3SI.

### Purification of Exosomes

Exosome-free medium was generated by centrifugation at 100,000 g overnight. Naive OT-I cells were seeded and incubated for 1 or 3 days, and then the cell supernatants were harvested for exosome purification. Briefly, cells were centrifuged at 300 g for 5 min remove cells and followed by 2,000 g for another 30 min to remove debris. The supernatant was collected and filtered through a 0.22 μM filter (Corning, NY). Exosomes were precipitated by PEG6000 (Millipore, Darmstadt, Germany) overnight and pelleted by ultracentrifugation at 100,000 g twice for 70 min at 4°C (Beckman Optima XPN-80, Beckman Coulter, Indianapolis, IN). The pellets were collected and washed with cold 1XPBS and followed by ultracentrifugation at 100,000 g twice for 70 min at 4°C (Beckman Optima XPN-80, Beckman Coulter, Indianapolis, IN). Purified exosomes were examined for protein concentrations by Commassie plus Protein Assay Reagent (Thermo Scientific, Rockford, IL) and stored at −80°C until use. Size distribution of exosomes was estimated by a Malvern Zetasizer Nano ZS90 (Malvern, UK) (48).

### Preparation of Cellular Fractions From 2SI- or 3SI-Stimulated CTLs

Freeze-thaw lysis and sonication were used (52–54) 10 million 2SI- or 3SI-stimulated CTLs were harvested as a cell pellet three days after each stimulation. Each cell pellet was resuspended in 100 ul of cold 1xPBS, which was followed by three cycles of freeze-thaw on dry ice. To further disrupt cell structure, each sample was sonicated for 10 s on ice for six times with 30 s intervals between pulses at 20 kHz on a sonicator 350 (Plainview, NY). The treated sample was then resuspended into 5 mL (total volume), centrifugated at 2,000 g for 30 min at 4°C. The pellet was resuspended in 100 μL 1xPBS and labeled “debris.” Ten μL of “debris” was added to each well (96-well plate). The supernatant was filtered with a 0.22 μm syringe filter (GVS, Sanford, ME), and flow through was collected and labeled as “soluble fraction.” Protein concentrations were determined using BCA assay (48).

### Exosome Functions on Low- or High-Affinity, Peptide-Stimulated CTLs

Flat-bottom Microtiter plates (Greiner bio-one, Frickenhausen, Germany) were coated only with recombinant MHC I (DimerX H-2Kb: Ig fusion protein). The coated plates were pulsed with altered N4 peptides of different affinities, N4, A2, Y3, Q4, T4, and V4 (high to low affinity) (12). Purified naive OT-I CD8+ T cells were placed at 5 × 10^4^ cells in 200 μL Allos medium in each well in a 96-well plate, with IL-2 at 2.5 U/mL, unless indicated otherwise. Purified exosomes were added into wells either at the beginning of or after stimulation at the indicated times, at a concentration of 33 μg/mL as used in human T cells (55) and our previous report (48). Cells were harvested for assay at the indicated times after stimulation.

### Transmission Electron Microscopy

Exosomes containing 0.03–0.3 ρg protein were suspended in 2% glutaraldehyde, applied to a Formvar-coated grid, and negatively stained with uranyl acetate. Electron microscopy was performed using a Zeiss EM10 transmission electron microscope at an accelerating voltage of 80 keV 26, as described previously (48).

### Western Blot

An aliquot of 10 μg exosome proteins was separated by electrophoresis on a 10% SDS-polyacrylamide gel and analyzed by Western blot (48). First, the proteins were transferred to a polyvinylidene difluoride (PVDF) membrane (BioRad, Hercules, CA) and blocked with 20 mM Tris-HCl, 150 mM NaCl, 0.05%, and pH 7.6 Tween-20 blocking solution (TBST) containing 1% bovine serum albumin (BSA). The membrane was incubated with the first antibody at room temperature (RT) for 1 h and then washed 3 times using TBST to remove excess antibody. The membrane was then incubated with horseradish peroxidase (HRP)-conjugated secondary antibody for 1 h at RT. Signals were detected with a SuperSignal West Pico Chemiluminescent Substrate (Thermoscientific, Rockford, IL) and a Gel Doc imaging system (Biorad, CA).

### Cell Proliferation

Purified naive OT-I CD8+ cells were washed in Hank's Balanced Salt Solution (1 × HBSS) (Corning, Manassas, VA) and resuspended in 1 x HBSS at 1 × 10^7^ cells/mL containing carboxy-fluorescein diacetate succinimidyl diester (CFSE) for a final concentration of 0.5 μM and incubated for 5 min at 37°C before being transferred to cold Allos. Cells were then washed twice with Allos before plating (48).

### Cell Staining and T Cell Activation Analysis

T cell activation markers were examined by flow cytometer (BD Biosciences, San Diego, CA) and analyzed using FlowJo software (FlowJo, Ashland, OR) (48); markers included CD25, IFNγ, and GZB. IFNγ expression was induced by incubating cells in RP-10 with 0.2 μM OVA_257−264_ peptide and 1 μL Brefeldin A (BioLegend, San Diego, CA) for 3.5 h at 37°C. Cells were then fixed with 4% fixing buffer at a 1:1 ratio for 15 min at 4°C, followed by permeabilization in saponin-containing Perm/Wash buffer (Biolegend, San Diego, CA) for another 15 min at 4°C.

### Killing Assay

The CellTiter-Glo® (CTG) killing assay is based on the number of viable cells left in the culture after cytotoxic T lymphocyte killing of the target cells (56). B16.OVA melanoma cells adhere to plastic surfaces and can efficiently present OVA_257−264_ peptide; activated OT-I T cells recognize H-2K^b^/OVA_257−264_ and initiate specific killing of these B16.OVA cells (57, 58). B16.OVA cells were seeded onto 96-well white plates at 30,000 cells/well in 100 uL Allos medium, and activated OT-I cells were added to each well as effectors to target cells (B16.OVA cells) at a ratio of 10:1. After overnight incubation, T cell suspensions (both OT-I cells and B16.OVA) were removed by washing three times with Allos medium. Luminescent signals (relative luminescent unit, RLU) from a 96-well plate was measured by the addition of 200 μL of 50% Cell Titer Glo (Promega, Madison, WI) followed by measurement of luminesce using a plate reader (Bio-Rad). The kill percentage of the B16.OVA cells by effector OT-I cells was calculated according to the following equation: Killed % = 100% × (RLU of untreated B16.OVA cells—RLU of B16.OVA cells cultured with OT-I cells)/RLU of untreated B16.OVA.

### Proteomics

Exosomes containing 10 μg of proteins were incubated in 8 M urea at room temperature to disrupt their membranes. *Tryptic digestion* Exosomal proteins were reduced with DTT, alkylated with iodoacetamide, and digested with 0.5 μg Trypsin/LysC Mix (Promega, Madison, WI) at 35°C, first at 4 M for 1 h, then further diluted with 0.8 M urea to activate the trypsin and incubated overnight. Tryptic digests were acidified with 2 μL TFA and desalted with C18 TopTip (Glygen Corp., Columbia, MD). Eluted peptides were vacuum-dried and dissolved in 35 μL solvent A (2.5% ACN, 0.1% formic acid in water). Peptide concentration was estimated using a Qubit 3.0 Fluorometer. LCMSMS analysis was carried out using a Dionex U3000 nanoHPLC system interfaced to a Thermo Scientific orbitrap Fusion Lumos mass spectrometer. Samples were analyzed in randomized order with a solvent blank between samples. For each sample, 1 μg of tryptic digest was injected into an Accalaim™ PepMap™ 100 trap column (5 μm, 100 Å, 300 μm × 5 mm), and desalted at 5 μL/min with 100% Solvent A for 5 min. The peptides were then separated with an Accalaim PepMap™ 100 nano column (3 μm, 100 Å, 75 μm × 250 mm) using a linear gradient of 2–52% solvent B (75% ACN, 0.1% formic acid) over 160 min. Precursor masses were detected in the Orbitrap at R = 120,000 (m/z 200). Fragment masses were detected by linear ion trap at unit mass resolution. Data dependent MSMS was carried out at a cycle time of 3 s. Dynamic exclusion was at 30 s. Protein identification and label-free quantification were carried out using Proteome Discoverer software (v. 2.2, Thermo Scientific), and mouse proteome was downloaded from Uniprot (uniprot.org) using both Sequest HT and Mascot search engines. M oxidation and NQ deamidation were set as variable modifications, and carbomidomethylation of C was set as the fixed modification. Precursor mass tolerance was 20 ppm, later filtered to 5 ppm in the report. Fragment mass tolerance was 0.6 Da.

### Enriched Pathway Analysis of Differentially Expressed Genes

Data were uploaded into the Ingenuity Pathways Analysis (IPA) software (Ingenuity Systems, http://www.ingenuity.com). The IPA database is maintained and edited by humans and contains genes, proteins, and RNA not only to find associations between expression data and canonical pathways but also build new networks. The significance of associations was computed using the right-tailed Fisher's exact test. All signaling pathways identified by IPA with a *P*-value of less than 0.05 have a statistically significant, nonrandom association.

### Statistical Analysis

We used an unpaired, two-tailed Student's *t*-test in GraphPad (Prism 5.0 software; GraphPad Prism, La Jolla, CA, USA) for statistical analysis of significance.

## Results

### Exosomes Preferentially Enhance CTL Proliferation Stimulated by Low-Affinity Peptides

**Figure 1:**
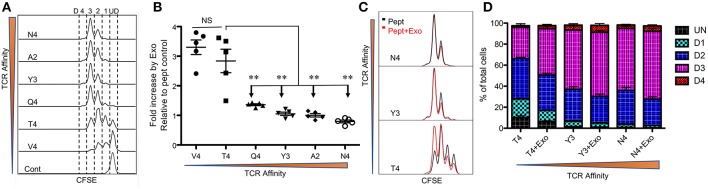
Exosomes preferentially enhance CTL proliferation stimulated by low-affinity peptides. Purified naive OT-I cells were labeled with CFSE and stimulated with peptides in the presence or absence of Exo. Peptides ranged from high affinity (peptide N4) to low affinity (peptide V4). **(A)** Representative CFSE dilution in OT-I cells stimulated for 2 days by peptides with diverse affinities presented by plate-bound recombinant MHC I (Dimer X). **(B)** Fold change caused by CTL-derived exosomes (Exo) in the cell number of OT-I cells compared to peptide-only controls. Comparisons were based on Student's *t*-test between T4 and each of other peptides. **(C)** Representative histograms. Black lines: peptide only. Red lines: peptide plus Exo. (ND: undivided cells; D1: cells divided once; D2: second division; D3: third division). **(D)** Effects of Exo on divisions of OT-I cells stimulated by different affinities. Data in **(A,C)** are representatives of at least 5 experiments. Asterisks indicate statistical significance. *P < 0.05; **P < 0.01; ***P < 0.001 by unpaired, two-tailed Student's *t*-test, which will be the same in the rest of this study.

Peptide/TCR affinity is the main contributor to CTL responses, so we first tested the effects of CTL-derived exosomes on OT-I cells stimulated by peptides of different affinities. Low-affinity peptides alone were able to drive the proliferation of naive CTLs, albeit at a slower rate (Figure 1A), consistent with previous reports (6, 13, 48). OT-I T cells treated with intermediate- and higher-affinity peptides (peptides N4, A2, Y3, and Q4) underwent similar rounds of cell cycles after a 48 h stimulation period. Low-affinity peptide stimulation (peptides T4 and V4) resulted in fewer cells entering the cell cycle; most remained undivided with peptide V4 (Figure 1A). The presence of exosomes (Exo) from the supernatant of fully-activated CTLs (48) led to a 2- to 3-fold increase in final cell numbers following 2 days of stimulation with low-affinity peptides (T4 and V4) in comparison to corresponding peptide-only controls; this was not recapitulated with intermediate or high peptide affinities (Figure 1B). These final differences from low-affinity peptides were similarly reflected by CFSE dilution. The addition of Exo to low-affinity peptide T4 stimulation drove OT-I cells into further divisions than T4 alone, whereas Exo did not change significantly in the dividing of CTLs stimulated by intermediate or high affinity peptides Y3 and N4 (Figure 1C). Moreover, Exo pushed more cells into the cell cycle during low-affinity peptide (T4) treatment, and more of these dividing cells entered division 3 by reducing the number of cells in division 1 (Figure 1D). Thus, Exo preferentially enhanced the proliferation of CTLs stimulated with low-affinity peptides, with no such effect on intermediate- or high-affinity, peptide-stimulated cells.

### Exosomes Preferentially Enhance the Expression of IFNγ in Low-Affinity CTLs

**Figure 2:**
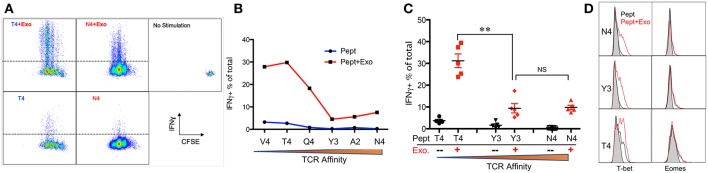
Exosomes preferentially enhance the expression of IFNγ in low-affinity CTLs. Naive OT-I cells were labeled with CFSE and stimulated by peptide with or without the Exo for 2 days. **(A)** Representative dot plots and gating for IFN-γ expression. **(B)** Representative data on the production of IFN-γ from a set of peptides with altered affinities. **(C)** Pooled data from multiple experiments on the production of IFN-γ. **(D)** Representative histograms of T-bet/eomes expression affected by Exo. Black lines: peptide only. Red lines: peptides plus Exo. Statistics were based on Student's *t*-test. Data in **(A,B)** are representatives of at least 5 experiments.

**Figure 3:**
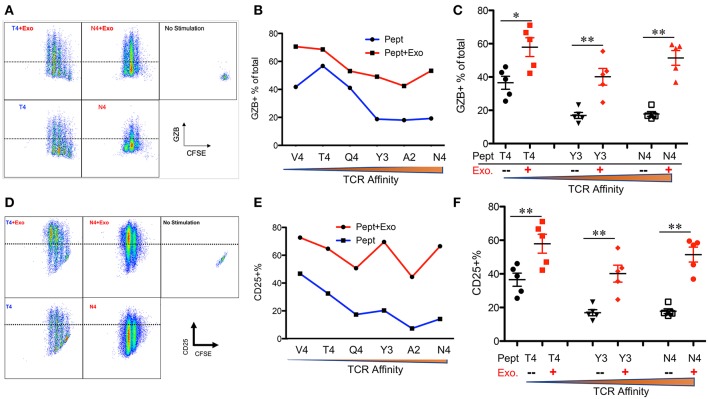
Exosomes induce the strongest activation in low-affinity CTLs. Naive OT-I cells were labeled with CFSE and stimulated with peptide with or without Exo for 2 days. **(A,D)** Representative dot plots and gating for granzyme B (GZB) **(A)** and CD25 **(D)** expression. **(B,E)** Representative data on the production of GZB **(B)** or CD25 **(E)** from a set of peptides with altered affinities. **(C,F)** Pooled data from multiple experiments on the production of GZB **(C)** and the expression of CD25 **(F)**. Statistics were based on Student's *t*-test. Data in **(A,B,D**,**E)** are representatives of at least 5 experiments.

IFNγ is critical for early protection against infections (59–62) as well as important for CTL function (63–65). IFNγ was barely detectable in any of the peptide-only treatments (Figure 2B), consistent with the fact that IFNγ production requires the presence of third signal cytokines such as IL-12 (50, 65). The presence of Exo slightly elevated IFNγ production in high affinity-stimulated CTLs (Figures 2A,B). Interestingly, IFNγ production increased when affinity decreased, with the highest production occurring in response to low-affinity peptides T4 and V4 (Figures 2B,C), suggesting a functional preference of Exo for low-affinity CTLs. Exosomes have been reported to mediate transcription signals during the immune response (66). In all treatments with Exo, the expression of T-bet (67) increased, with the most significant increase in the presence of low-affinity peptides; no effects were observed on eomes expression (Figure 2D). Exo can therefore preferentially enhance IFN-γ production in low affinity-stimulated CTLs, and this is positively associated with T-bet expression. However, this preference was not the case in GZB regulation by exosomes. First, GZB production was higher in low affinity-stimulated CTLs than in high affinity-stimulated CTLs (Figures 3A,B), consistent with a recent report using an *in vivo* system (27). Second, Exo increased GZB production at all affinities, with low-affinity-stimulated CTLs producing the highest total amounts of GZB (Figures 3B,C). In addition, killing ability was generally low for all affinities, and the addition of Exo enhanced the killing ability slightly, but significantly, only in low affinity-stimulated (T4) OT-I cells (Supplementary Figure 1). The expression of CD25 in low affinity-stimulated OT-I cells was higher than that in high-affinity stimulated OT-I cells, and the addition of Exo increased CD25 expression at all affinities (Figures 3D,E), without significant differences among them (Figure 3F). It thereby seems that exosomes preferentially enhance the activation of low affinity-stimulated CTLs.

### Exosomes From Partially Activated CTLs Fail to Activate Low Affinity-Stimulated CTLs

**Figure 4:**
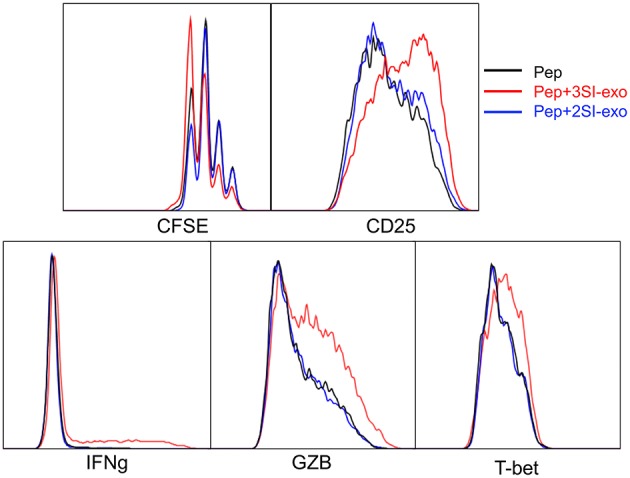
Exosome effects require three signal stimulation in secreting cells. Naive OT-I cells were labeled with CFSE and stimulated with peptide with or without two different types of exosomes for 2 days. 3SI-exo or 2SI-exo: exosomes secreted by 3SI-stimulated or 2SI-stimulated CTLs (48). Data are representatives of at least 4 experiments.

We found that exosomes derived from 2SI stimulation (antigen + costimulation) did not activate bystander CTLs (48). To test if these 2SI-exo had any effects on low-affinity stimulated CTLs, purified OT-I cells were stimulated with low-affinity peptide T4 in the presence or absence of 2SI-exo or 3SI-exo for 2 days. In contrast to 3SI-conditioned exosomes (3SI-Exo, the same as Exo), 2SI-exo had no effect on low-affinity-stimulated CTLs, demonstrated by unaltered expression patterns of IFNγ/GZB/CD25/T-bet, and instead appeared to inhibit their division (Figure 4). IL-2 is important for CTL activation (68, 69). To test if IL-2 played a role in exosome (3SI-exo) effects, purified OT-I cells were stimulated with low-affinity peptide T4 in the presence or absence of Exo and/or IL-2-neutralizing antibodies (70). The depletion of IL-2 completely abolished the effects of Exo as demonstrated by diminished expression of IFNγ/GZB/CD25 to a level even below T4-only controls (Supplementary Figure 2), suggesting that IL-2 is important to both peptide stimulation and exosome effectiveness. Of interest, despite the fact that IL-2 neutralization greatly reduced CTL activation at Y3 (intermediate affinity) and N4 (high affinity), the cell cycle progression was suppressed but not stopped (Supplementary Figure 2), suggesting IL-2 may not be definitely required for the cell cycle progression of CTLs.

The standard protocol for exosome purification is based on differential centrifugation and participation, which should remove most cell debris, but small contamination is still possible. To test the effects of potential contaminants in purified exosomes, cell debris and soluble fractions, another potential contaminant in exosomes, from activated CTLs, were prepared and added to high- or low-affinity stimulated CTLs, at a high ratio (debris from 20 cells to one stimulated cell) or protein concentration (5-fold of exosomes). When CTLs were stimulated with high-affinity N4, cell debris from both 2SI and 3SI inhibited GZB production, with minimal effects on proliferation (Supplementary Figure 3). In low-affinity stimulation, GZB production was also decreased by the presence of cell debris, but while 2SI-debris seemed to inhibit CTL proliferation, 3SI-debris did not (Supplementary Figure 3). Surprisingly, the soluble fraction from 3SI-CTLs did enhance GZB production, but dramatically inhibited cell proliferation, in both high- and low-affinity stimulation conditions (Supplementary Figure 3). The effects of soluble fractions were dose-dependent and became undetectable at a concentration of 1/5 exosomes (Supplementary Figure 4). These data suggest that the effects of potential contaminants are different from the effects of exosomes, and the level of contamination is unlikely to be as high as was tested in this experiment. Thus, the functions of exosomes (Figures 1, 2) are likely to be due to the exosomes, not potential contamination from exosome-producing cells.

### CTL-Derived Exosomes Enhance Early Activation of Low-Affinity-Stimulated CTLs

**Figure 5:**
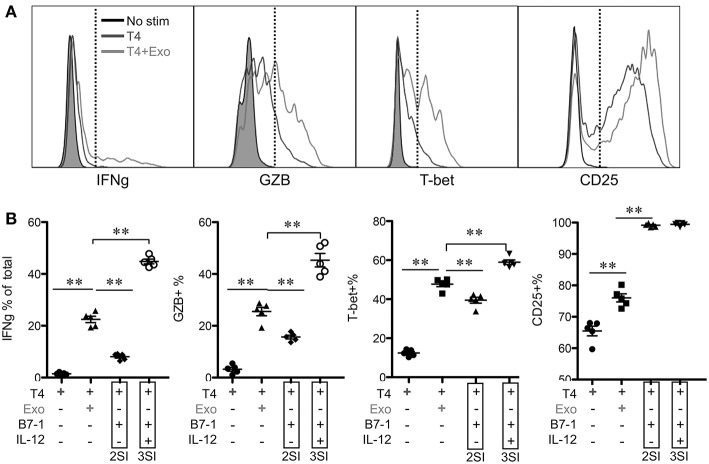
CTL-derived exosomes enhance early activation of low-affinity stimulated CTLs. Naive CTLs were stimulated with low affinity peptide T4/MHC I with or without Exo (3SI-Exo). Cells were analyzed 24 h after stimulation. 2SI: T4/MHC I+B7-1 (50). 3SI: T4/MHC I+B7-1+ IL-12. Statistics were based on Student's *t*-test.

To test if the activation of low affinity-stimulated CTLs was accelerated by CTL-derived exosomes, Exo was provided at the beginning of stimulation. IFNγ and GZB, which were undetectable at 12 h, were enhanced by Exo after 24 h (Figure 5 and data not shown). Most of these IFNγ-producing cells were also GZB+ (data not shown). The positive control peptide T4+costimulation (B7-1)+IL-12 (3SI) resulted in the highest production of both molecules at 24 h, whereas T4+costimulation (B7-1) (2SI) only induced low levels of GZB and IFNγ, suggesting that third signal cytokines may contribute to the activation of low-affinity CTLs in a similar pattern to but at lower levels than high-affinity CTLs (50, 65). Interestingly, T-bet was detectable in T4-stimulated CTLs and enhanced by Exo to a level close to that of 3SI (Figure 5), suggesting that this IFNγ enhancement by Exo may be regulated through T-bet (71, 72). CD25 expression was also increased by Exo, although this enhancement was only detectable after 24 h (Figure 5 and data not shown). Interestingly, CD25 expression peaked at 24 h (Figure 5), and started to decline at 48 h after stimulation (Figure 4). These data demonstrate that CTL-derived exosomes can enhance the early activation of low affinity-stimulated CTLs in a pattern similar to 3SI.

### Low-Affinity CTLs Remain Responsive to Exosomes for a Prolonged Period of Time

**Figure 6:**
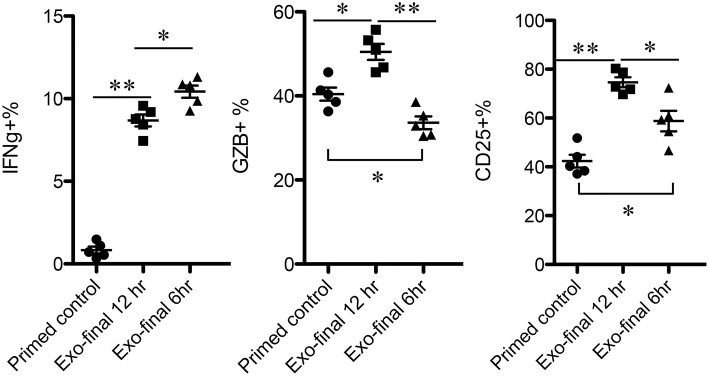
Low-affinity-primed CTLs are sensitive to CTL-derived exosomes. Naive CTLs were stimulated with low-affinity peptide T4/MHC I for a total of 72 h. Exosomes were added to stimulated cells at 60 or 66 h after stimulation, indicated as final 12 or 6h.

The time points of available Exo for low-affinity-stimulated CTLs may vary *in vivo*. To examine how low-affinity primed CTLs respond to Exo, primed CTLs were exposed to Exo at different time points after priming. Exo were added for the final 6 or 12 h during a total incubation time of 72 h, and exosomes added at the last 12 h were able to enhance expression of IFNγ, GZB, and CD25 (Figure 6), although to lower levels of IFNγ compared to a full 48-h exposure (Figure 2). Interestingly, exposure to exosomes for just 6 h enhanced IFNγ, but decreased GZB, suggesting differential responses to exosomes by low-affinity CTLs primed at different times. Nevertheless, the swift responsiveness of primed CTLs suggests that CTL-derived exosomes may perform posttranscriptional regulatory functions, possibly via regulatory or signaling proteins.

### Activated CTLs Secrete Exosomes at Early Stages of Activation

**Figure 7:**
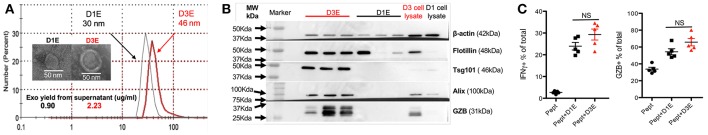
CTLs secrete functional exosomes soon after 3SI stimulation. Exosomes were purified from 24-h (D1E) or 72-h (D3E) supernatant of CTLs stimulated with 3SI (MHC I/N4+B7-1+IL-12). **(A)** Representative size distribution and electron microscopy of 3 experiments. **(B)** Western blot based on 10 μg protein of exosomes from 3 different experiments. **(C)** Naive OT-I cells were stimulated with low-affinity peptide T4 with or without D1E or D3E for 2 days before staining.

We next explored how quickly high-affinity CTLs could produce exosomes after being activated. Naive OT-I cells were stimulated with 3SI (with high-affinity peptide N4) (48, 50) and the supernatant was harvested for exosome isolation at days 1 (24 h) and 3 (72 h) after activation. Exosomes were smaller at day 1 (D1E) than at day 3 (D3E) (Figure 7A). Most classical exosomal proteins were not detectable in D1Exo, such as Tsg101 and Alix, while flotillin was detected at variable levels among the different batches (Figure 7B). The yield of exosomes in D1E was about 40% that of D3E (Figure 7A), but time for exosome production was two-thirds shorter. The D3E were derived from many more cells (8- to 10-fold after massive division) than the D1E from undivided CTLs. Thus, early-activated effector CTLs produced more exosomes than late effectors on a per cell basis. Importantly, D1E induced similar levels of IFNγ and GZB as D3E in low affinity-stimulated CTLs (Figure 7C). It appears that exosome secretion can begin soon after activation, and despite their differences in protein profiles (Figure 7B), D1E and D3E were similarly effective in enhancing the function of low affinity-stimulated CTLs.

### Early and Late CTL-Derived Exosomes Share Effector Functions Based on Protein Profiles

We examined the protein profiles of exosomes derived from CTLs activated in the presence of IL-12 together with antigen/costimulation for either 24 h (D1E) or 72 h (D3E) (Figure 7). A total of 2097 proteins were identified from three biological replicates at each time point (Supplementary Table 1). Despite the fact that most proteins were detected in both exosome populations, there were substantial quantitative differences. Using 1.5-fold as the cutoff, 467 proteins were enhanced (or unique) in D1E (Supplementary Table 2), and 233 in D3E (Supplementary Table 3). We next explored how these skewed (differential) protein profiles were associated with downstream effector function using Ingenuity Pathway Analysis (IPA) (73). The two different profiles were predicted to relate to similar biological functions, such as enhancing the activation, movement of cells, inhibiting apoptosis, and cell death (Table 1). This result is consistent with functional data (Figure 7) and suggests that the exosomes generated by early and late effectors may contain different proteins but induce similar functions.

**Table 1:** Predicted shared effector functions of differential exosomal proteins from CTLs stimulated for 1 and 3 days *in vitro*.

**Functions**	**Exo**	***P*-value**	**Molecules**
Apoptosis ⇓	D1E	2.07E-08	64
	D3E	5.22E-18	78
Cell death ⇓	D1E	1.32E-18	97
	D3E	3.05E-29	69
Movement ⇑	D1E	1.41E-20	80
	D3E	6.98E-12	85
Activation of cells ⇑	D1E	4.92E-07	31
	D3E	1.52E-08	49

*Differential proteins either in D1E (Supplementary Table 2) or D3E (Supplementary Table 3) were analyzed using Ingenuity Pathway Analysis (IPA)*.

## Discussion

Low-affinity CTLs are critical components of the immune response. We found that fully activated CTLs can secrete exosomes that preferentially enhance the activation of low-affinity CTLs, suggesting potentially interconnected communication between fully activated, high-affinity CTLs, and low-affinity CTLs through exosome secretion with several noticeable features. First, the original exosome-secreting CTLs required 3SI stimulation; 2SI stimulation-induced exosomes failed to enhance the activation of low-affinity CTLs. Second, this communication begins early, as a large quantity of functional exosomes were secreted shortly (24 h) after 3SI stimulation. Third, although we cannot exclude the possible function of shared common molecules in both early and late CTL-derived exosomes, pathway analysis suggests that the differential proteins between exosomes from these two stages share common downstream effector functions. Finally, low-affinity CTLs can respond to exosomes for a prolonged period of time.

Although studied in a simplified *in vitro* system in this project, these data may recapitulate similar communication among CTLs of different affinities in animals. The output by the thymus of naive CTLs with varying affinities is a continuous process; thus, they may not be activated at the same time. Infection alters inflammation profiles and kinetics, and the necessary third signal cytokines may not be equally available to CTLs in different tissues (74). Low-affinity CTLs may also be exposed to exosomes at different time points after priming. Our data suggest a potential communication through exosome secretion between fully activated, high-affinity CTLs and low-affinity CTLs, which needs to be further tested in animals.

Exosomes can be detected at relatively high concentration (0.1–20 × 10^9^ particles/mL) in human plasma (75–77), with many potential sources such as epithelial and immune cells (78, 79). Mature mouse dendritic cells can produce exosomes at about 10 μg/mL in the supernatant after 48 h culture (80). After stimulation with 3SI for 1 day, CTLs can produce exosomes at 0.9 μg/mL in their supernatant from 3 × 10^5^ cells (Figure 7A), equal to 30 μg/mL based on 10^7^ cells, suggests that activated CTLs may be an important source of exosome production. Exosomes have been mostly investigated as biomarkers for diagnosis and drug delivery (78, 79), but recently, some clinical trials have been carried out to test the function of *in vitro*-generated exosomes in cancer patients, using relatively low doses based on the number of MHC II molecules detected in exosomes (81, 82). Despite differences among disease models (82), preclinical experiments in mice mostly used an exosome dose in the range of 0.1–50 μg/mouse, administered mostly subcutaneously; a higher dose may be required for a dermatology model (82). Nevertheless, it seems CTL-derived exosomes may be important under certain physiological conditions, which we will further examine in animals.

The mechanisms that underlie the preference of CTL-derived exosomes for low-affinity CTLs are not known. TCR signaling triggered by weak ligands appears different from that triggered by strong ones (43) and cannot be explained by dose effects (44, 45). One explanation may be the skewing of low-affinity CTL activation toward a profile similar to 3SI stimulation, but different from high-affinity stimulation. Mechanisms for primed low-affinity CTLs may also be time-dependent. Initial exposure to exosomes induced an activation status in primed low-affinity CTLs resembling 3SI stimulation, whereas late exposure led to enhanced IFNγ but reduced GZB expression. These data indicate, at least conceptually, that the mechanisms for exosome activation of low-affinity CTLs may not be a simple switch from A to B, but rather a status-based response potentially driven by different exosomal proteins. In addition, despite the similarity in size and expression of exosomal markers, exosomes are generally heterogenous (82); thus, the effects could be induced by different proteins in the same exosomes, or, different exosomes from the same population. Pathway manipulation, at least *in vitro*, may be useful in narrowing down the number of potential targets; this is ongoing in our lab.

Exosomes from fully activated CTLs can preferentially enhance the function of low-affinity CTLs to a certain level, but not to the level of fully activated CTLs stimulated by 3SI. This was demonstrated by activation markers, effector molecule expression, and killing ability assays. We speculate two possible functions for exosome-facilitated communication between high-affinity and low-affinity CTLs. Either this communication can transform a functional heterogenous CTL population against one pathogen or cancer to a more homogeneous population, or exosomes secreted by fully activated CTLs may not influence direct killing but rather support general immune regulatory function through critical factors like IFNγ, thus affecting multiple cell types, including CD4 T cells.

It is important to note that the presence of IL-2 is required for the function of exosomes, with implications for the initiation of the immune response and autoimmune disease. During infection or an anti-tumor immune response, strongly activated CTLs in the presence of IL-12 and IL-2 will secrete exosomes quickly to promote the function of a robust population of low-affinity CTLs. When IL-2 is no longer available, such as when the infection is resolved, exosomes can no longer affect low-affinity CTLs. If fully activated CTLs can be induced to secrete functional exosomes in humans in ways similar to mice, these functional exosomes could be injected together with IL-2 to boost low-affinity CTLs in immunotherapy for chronic infections and cancers. T cell-derived exosomes are found to be able to induce the production of inflammatory factors such as IL-6, IL-8, and MCP-1 (83). Because most autoantigens target low-affinity CTLs, the secretion of stimulatory exosomes from fully activated CTLs is a legitimate concern in the induction of autoimmunity. Based on the definitive IL-2 requirement for the function of exosomes on low affinity CTLs, we speculate that this concern is limited to the period of IL-2 production. Conversely, if CTL-derived exosomes are indeed involved in autoimmune disease, neutralization of IL-2 could become an effective method to dampen exosome function.

Communication between high-affinity and low-affinity CTLs might occur through secretion of different exosomes. Cells may adopt different methods to generate and protein-load exosomes in response to diverse environments. Although both early and late stimulations can induce abundant exosome production in CTLs, the sizes of the vesicles and types of proteins contained therein differ noticeably, suggesting that the duration of stimulation directs both exosome formation and its content. The production of exosomes follows one of two pathways, ESCRT (Endosomal Sorting Complex Required for Transport)-independent or ESCRT-dependent (84) modalities. Tsg101 and Alix are major components of the ESCRT-dependent pathway (85). A lack of these proteins in early exosomes suggests that early-stimulated CTLs might utilize ESCRT-independent pathways to generate exosomes, whereas late exosome formation in CTLs follows a more measured, ESCRT-dependent route. It is also not clear if the late-isolated exosomes represent an accumulation of total exosomes from early and late stimulations. Further experiments will be necessary to unpack specific mechanisms of CTL exosome secretion at different time points.

**Figure 8:**
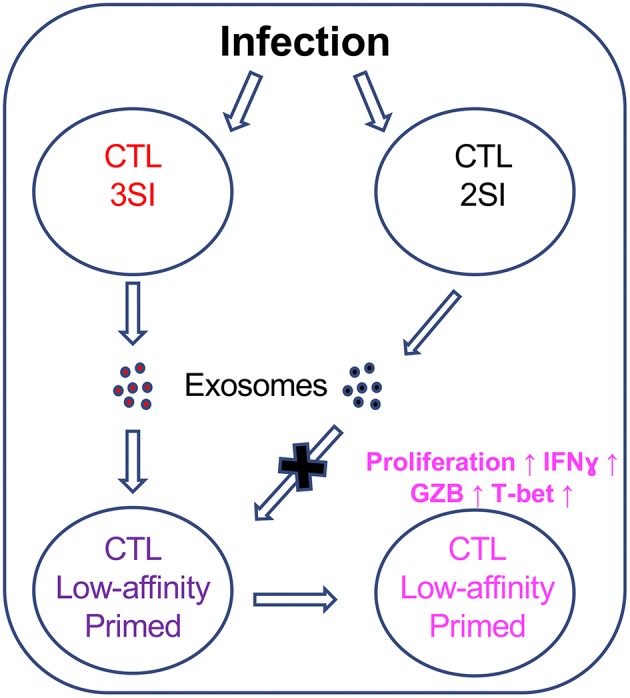
CTL-derived exosomes enhance the activation of CTLs stimulated by low-affinity peptides. Fully activated (3SI) CTLs secrete exosomes capable of enhancing the activation of low-affinity CTLs, whereas partially activated (2SI) CTL-derived exosomes were not able to do so.

While there are undoubtedly many factors involved in the initiation and maintenance of the low-affinity CTL immune response, our data indicate that exosomes secreted by fully-activated CTLs could preferentially enhance the activation of CTLs stimulated by low-affinity peptides, thus providing the first evidence that CTL-derived exosomes could contribute to a previously unappreciated communication between fully activated, high-affinity CTLs and low-affinity CTLs (Figure 8).

## Ethics Statement

Mice were maintained under specific pathogen-free conditions at the University of Maryland, and these studies have been reviewed and approved by the Institutional Animal Care and Use Committee.

## Author Contributions

ZX conceived the study and coordinated the study. ZX, S-WW, YW, and LL designed, performed, and analyzed the experiments and wrote the manuscript.

### Conflict of Interest Statement

The authors declare that the research was conducted in the absence of any commercial or financial relationships that could be construed as a potential conflict of interest.
