# Supplementary material for: CTL-Derived Exosomes Enhance the Activation of CTLs Stimulated by Low-Affinity Peptides
Source: Front Immunol. 2019 Jun 4;10:1274. doi: 10.3389/fimmu.2019.01274 (PMC6593274; doi:10.3389/fimmu.2019.01274)
Supplement: Supplementary file 1 [file Data_Sheet_1.PDF]

CTL-derived exosomes enhance the activation of CTLs stimulated by low-affinity peptides

Shu-Wei Wu[1], Lei Li[1], Yan Wang[2] and Zhengguo Xiao[1]

[1]Department of Animal and Avian Sciences, University of Maryland, College Park, Maryland 20742, USA
[2]Department of Cell Biology and Molecular Genetics, University of Maryland, College Park, Maryland 20742, USA;

**Corresponding Author:** Zhengguo Xiao, Ph.D.
Department of Animal and Avian Sciences
University of Maryland
College Park, MD 20742 USA
Phone: 301-405-6258
FAX: 301-405-7980
Email: xiao0028@umd.edu

**Index for supplementary information**


| Information | Pages |
|---|---|
| Suppl.Fig.1 Exo enhances the killing ability of low-affinity stimulated CTLs | 1 |
| Suppl.Fig.2 IL-2 is required for the effects of Exo | 1 |
| Suppl.Fig.3 Cell debris does not enhance the activation of CTLs stimulated by high- or low-affinity | 2 |
| Suppl.Fig.4 Effects of soluble fractions (SF) are dose-dependent | 2 |
| Suppl.table.1 Total proteins detected in D1E and D3E | 3-64 |
| Suppl.table.2 D1E differential proteins | 65-73 |
| Suppl.table.3 D3E differential proteins | 74-79 |

**Supplementary information**

**Manuscript: CTL-derived exosomes enhance the activation of CTLs stimulated by low-affinity peptides**

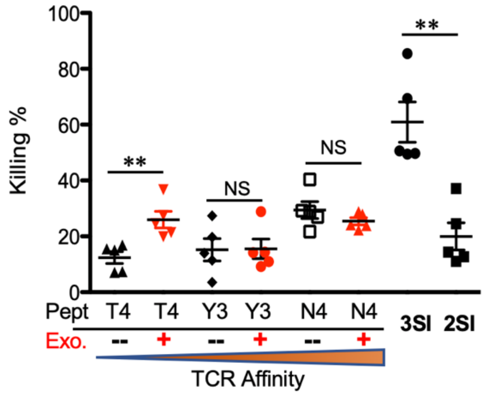


Supplementary Fig 1. Exo enhances the killing ability of low-affinity stimulated CTLs. Naive OT-I cells were stimulated with peptide with or without Exo, before seeded with B16.OVA at the ratio of 10 (effector) :1 (target). Killed % = 100% x (RLU of untreated B16.OVA cells – RLU of B16.OVA cells cultured with OT-I cells)/RLU of untreated B16.OVA. Data are representatives of 3 experiments.

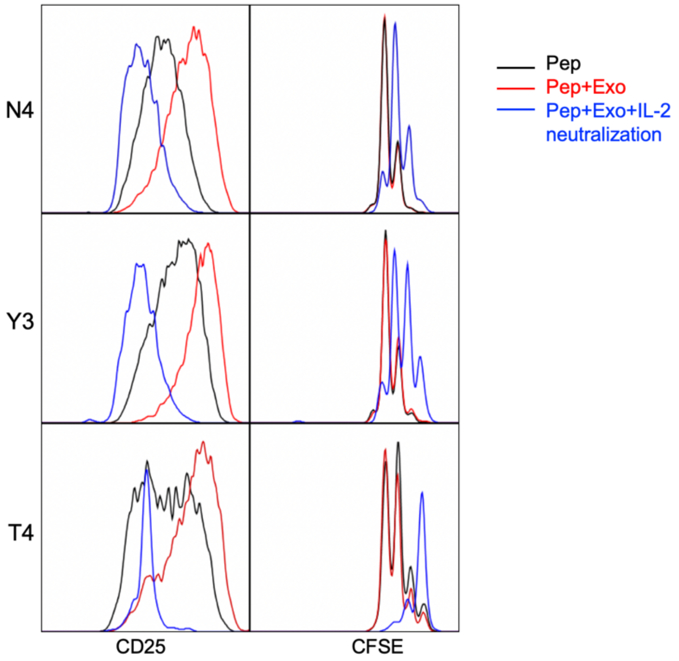


Supplementary Fig. 2. IL-2 is required for the effects of Exo. Naive OT-I cells were labeled with CFSE and stimulated with peptide with or without Exo or (Exo+IL-2 neutralizing antibody) for two days. Exo: exosomes secreted by 3SI-stimulated CTLs. Data are representatives of at least 3 experiments.

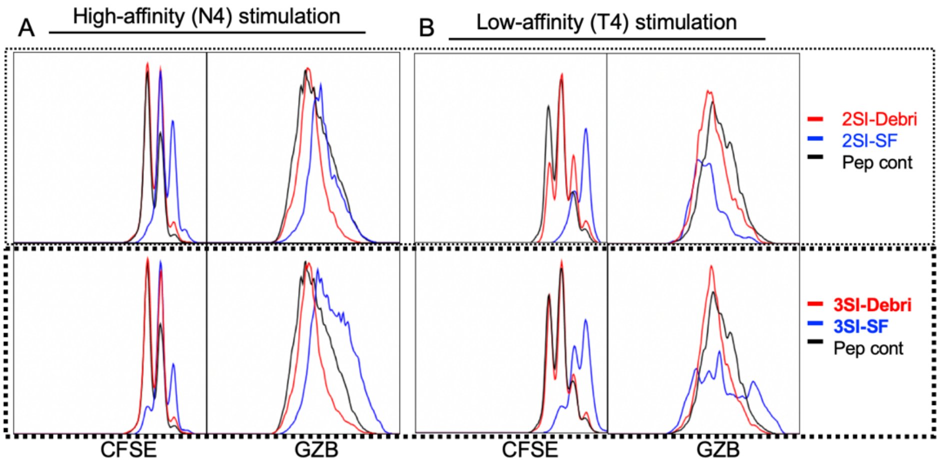


Supplementary Figure 3. Cell debris does not enhance the activation of CTLs stimulated by high- or low-affinity. Naive OT-I cells were cultured with dimer X coated plates pulsed by either N4 (A) or T4 (B) peptides in the presence or absence of cell debris at a ratio of 20 to 1 (debris from 20 activated CTLs was added to one stimulated CTL). Soluble fractions (SF) were similarly examined, at a protein concentration 5-fold of exosomes, which is 5 x 33 µg/mL =165 µg/mL in A-B, or at 165, 33, and 6.6 µg/mL in E. CTLs were harvested for staining two days after culture.

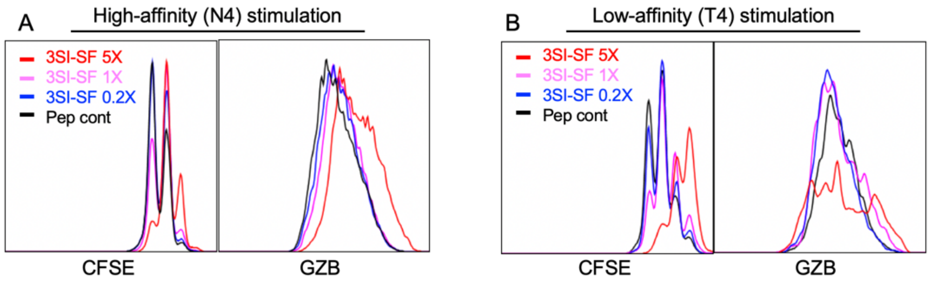


Supplementary Figure 4. Effects of soluble fractions (SF) are dose-dependent. Naive OT-I cells were cultured with dimer X coated plates pulsed by either N4 (A) or T4 (B) peptides in the presence or absence of soluble fractions (SF) at a protein concentration 5-fold (5X), 1-fold (1X) or 0.2-fold (0.2X) of exosomes, 165, 33, and 6.6 µg/mL respectively. CTLs were harvested for staining two days after culture. Data in Suppl. 3 and Suppl.4 were generated in the same experiment, but presented separately for the purpose of easy comprehension.

**Supplementary Table 1.** Total proteins detected in D1E and D3E. Three different biological replicates of D1E and D3E were analyzed by proteomic assay. The detection of protein was only validated if its presence occurred in all three replicated. The list of proteins contains all proteins identified in either D1E, D3E, or both.

| **ID** |
|---|
| G3UWX1 |
| Q545X8 |
| Q61598 |
| Q00796 |
| Q542S2 |
| Q9CXW3 |
| Q07954 |
| P08514 |
| P33610 |
| Q8CC86 |
| Q9CX34 |
| O70274 |
| Q8C3J5 |
| P12382 |
| Q04857 |
| Q61035 |
| P04180 |
| P30681 |
| Q61838 |
| Q9CQI6 |
| P35579 |
| Q9D662 |
| Q0PD50 |
| P12960 |
| Q7TNC4 |
| Q9CQ65 |
| P20774 |
| Q99JR5 |
| P08775 |
| E9QMV2 |

Q80UG5

P46531

Q91Z50

Q8K1M6

Q9D6Z1

Q8BLR5

Q80TH2-2

Q80XI4

Q9ES61

A3KG93

P62748

Q92859

O70251

O75891

Q80UF4

Q8BP67

P63321

P56873

Q8VDW0

P70452

Q80TY0

P62852

P01590

E9QLW5

Q6P8X1

P68134

P62264

O35235

Q9QZJ6

P02787

P27601

Q9CZ44-3

A2AMH4

Q9WVJ2

E9PXC0

Q96EK6

Q9R0N0

Q9DBG7

Q16772

Q3TZH4

P06858

Q92747

Q9JMA1

P21995

Q9ET30

P21550

S4R1S4

Q8BKX1

P14429

P50396

Q3UFS5

D9J302

Q9QUJ7

P68433

Q91Z38

A0A0R4J097

B1AZI6

Q921G6

B0V2N1

P24549

Q99JA5

Q544U7

Q9CYR6

P34022

Q3U1F9

O35864

Q543K5

Q8CGC7

Q8BHN5

Q5SUR3

Q3U1J4

Q58E49

Q6ZMU1

P99027

Q922I7

Q8VHR5

P62960

P47880

P04275

Q9ESX5

P13861

P08123

Q1HFZ0

P55264

P70429

Q9D1Q6

Q99JX4

A0A087WSP5

Q01102

E9QN31

Q8CFE6

Q9WVH9

E9QN99

Q5SWD9

Q3TRJ7

Q9QY81

Q93063

Q9WV80

P63017

Q1HL32

Q8K094

Q9WTI7

P17427

Q792Z1

Q3UM45

P53996

P02751

P07356

P40124

Q921F2

Q9WUL7

Q640N1

P15121

P23526

P43406

Q64487-10

Q60676

Q3TPZ5

Q9HDC9

Q3UPI8

Q76MZ3

G3UXA6

P28843

Q99PL5

I3L0U2

Q9CYL5

Q545B6

D3YTN4

Q3U429

A0A023T778

Q3UEB3

F6W687

Q91X52

Q04447

O35841

Q8BUM3

P62835

E9PYL9

P84228

P05533

Q543E3

Q542I2

Q6PD03

Q545C3

Q8CG48

Q8BG05

E9QM99

P38647

Q9D4H8

O54825

P60670

Q5EBP8

P62141

Q60692

Q9R1C7

Q8VD58

Q8VDZ4

O08582

Q12860

P70268-2

P80317

P01887

Q8CIB5

P01899

O88890

O88952

P08249

O35295

Q99JI4

P13639

Q62009

Q544Z7

P61226

P54289

E9PZQ0

Q62179

P26516

Q3ULL5

G3V4T6

Q8BJY1

P62855

P28301

P50296

Q9NZN3

Q811D0-3

P58854

P70195

Q9D2C2

Q60973

Q91ZW3

A0A0N4SUZ3

Q9Z2D6-2

G3X9B1

P16152

Q8R105

P10852-2

P31939

Q9CPP0

Q11011

O35593

A0A0R4J0I9

Q6ZWN5

Q3TEK8

E9Q3X0

P20065

Q9R190

J3QNG0

A0A0R4J0P5

Q8JZU2

E9QK48

Q20BD0

Q543N7

E9QNN1

Q8BFY6

Q3TM70

P27659

Q9DBZ5

P04925

E9QPX1

Q562R1

Q64287

A0A0R4J247

S4R270

Q9Z1R2

P97429

A2AQL0

P61255

Q9D0M5

O08638

Q924H7

P61020

P10404

A0A087WVP1

A8IP69

O08759

Q60769

Q61672

Q02242

Q9CS42

J3QPG5

Q9UBG0

A0A087WV75

P11717

Q8BMJ2

P42229

Q6ZQL4

P61222

P41105

O54734

F8VQJ3

Q9HD89

A0A0R4J088

O70624

A0A0R4J0S2

Q61081

P09793

P35123

Q02788

Q3U1N0

Q5SQX6

P60335

G3UXW9

Q9QYF9

Q9CXY6

P63085

P09242

P40336-2

P42208

O08912

P62267

A0A0R4J135

Q9Z1R9

P47226

Q6PGB6-4

A0A1B0GU03

G5E8S8

Q9WU78

P40142

P54923

Q91VH6

Q8BFQ3

Q3U2C5

H3BJL0

Q9QUR7

Q8BH57

A0A087WQT6

Q8VDF3-2

A6H6K1

Q0PD49

P39688-2

P84095

G3UYJ7

Q3ULW8

E9PW66

Q8CG47

Q9JKB3-2

Q3TC45

Q6P9R2

P97351

P54822

Q8C503

P28063

Q9CZU3

Q544T7

Q6AXD2

Q8K3H0

O55222

F8WIV5

P22352

Q9ES52

O35375

Q3U741

P07358

Q12805

P70372

Q61451

P07195

P62962

Q8K2Y9

P24270

Q8BVQ9

G5E870

E9PWG6

Q76LX8

Q9CWI3

Q9D0R8

P51150

P17742

P62081

Q8BGH7

A0A0R4J1V1

P61967

Q8BP47

Q8C147

H7BX95

Q9Z0H4-4

Q9DC11

Q91VR8

Q99LE1

A0A0R4J256

F8WJE0

Q9DAK9

A0A0G2JPR0

P16045

Q8CD15

O09126

P58021

Q8R1G2

Q8K4B0

P0C0A3

Q8C4J7

Q9QZH3

Q5M9L7

Q8VED9

P23198

E9PYH2

Q61098

A0A0B4J2C3

Q8K2H4

Q8R001

Q6PAC3

Q99M54

Q9QYJ3

Q01518

Q64462

O43776

P52594

O55143

Q9Z0N1

P28070

Q8VEJ9

P09417

Q6NS45

G3X9Q0

G3V5Z7

Q9CWJ9

Q9BRA2

Q05144

Q922Q8

Q9QWR8

P62889

P14733

P70336-2

Q3UNF2

Q62312

Q5M8R8

Q4VBE8

Q8R0Y6

Q9CR57

Q3UL22

P11983

E9QJS1

Q0PD35

P10630-2

Q3U4Y3

Q6R0H7

D3Z6X4

P62937

Q9Z1F9

Q7TMK9

Q9CZ13

H3BKH6

Q91Y47

P32921

P63242

B1B507

Q9D1M4

Q8VI75

Q9CQ71

P42209

Q6PDI5

P24527

Q9D0I9

A0A0R4J0B7

Q8BGM4

A2BFF9

A0A087WRE8

P62911

Q8VEH3

Q91XH5

P61626

Q7TQI3

Q99MI6

E9QK83

Q9CQV8

B2RTM0

Q64152

P15532

E9PV41

Q9QYI3

D3Z2H9

Q923F1

Q00609

Q9D7S7

Q9JJK2

E9PGA6

A2A8L5

P35285

Q2LA85

Q8BZA9

Q06828

Q91W89

Q71FD5

Q8BU31

F6RPJ9

G3X982

Q3TQX5

Q3TA56

Q6V0I7

P70352

Q9D0L8

Q9QZI9

Q8CGP2-2

Q99NH0

O09061

Q5M9M0

P54823

Q5BLJ9

Q91V89

Q3UE37

Q542B0

Q99KK9

Q3TD53

Q8BYZ1

P00375

Q99LE6

Q8C605

P30101

P15702

P23188

Q545R3

G3X956

P14602

A0A0A6YWR2

E9PZF0

P25444

E9QAS5

E9Q696

P70695

Q9CY21

K7EIK7

Q8BWY3

P80316

Q52KI8

Q8CGY8

O55226

P22682

Q6P2B1-2

G3V1V0

P06801

P49773

C9JC84

Q9JLZ6

Q9DB05

A0A087WR52

B2RQ80

P05556

P01751

Q542I9

Q8C7E4

D3Z4B2

A2AW86

A0A0A0MQA5

P50431

E0CX20

Q9D176

Q3UAD6

P97814

Q3TBT3-2

Q9WVA2

Q9CZX8

P61971

Q8BFY9-2

Q80T14

Q9UM47

A0A0R4J0J6

O14818

Q60605-2

P16330

Q8BWP8

P11688

A0A0U1RP94

P70697

P35527

Q07113

P53986

Q5SS40

Q9JKB1

E2JF22

P63101

Q80ZJ1

G3X977

P68036

Q149Z9

Q9QXS1

Q5EBQ6

Q543J5

E9Q7Q3

Q8BYA0

P05387

Q9WTP6

Q9CR26

O70293-2

Q9DBD5

Q9D8U8

O14786

P00488

Q60865

A2AN08-5

Q01965

Q6DFW4

Q6PHN9

P62482

Q61655

O35218

Q8BVY0

P07384

P32883-2

Q8R5K4

P70460

Q9CY58

Q5M9M4

Q7Z4W1

Q9JK38

Q9WVA3

Q8BPU7

P61089

Q9QYB1

Q8K285

Q6UY14

P52209

Q8VEK3

G3XA17

Q6PFB2

O70591

P63168

Q6P2I3

Q9WUP7

Q7TPR4

Q6IRU2

Q3ULF7

P08758

G5E8C3

Q922B1

Q8CI11

Q8BTM8

Q08288

Q546H1

Q9NPG4

Q9QZC7

P14780

P63163

Q9WTM5

A2RS22

E9PVB7

Q8BM72

P68040

K4DIA0

G5E924

Q4VA93

P47911

D3YX87

P13595

K7ERG9

P52431

Q8BU30

O09167

B9EHN0

O35344

P10493

Q8CI33

Q3THK7

P62196

Q3U4U6

P52480-2

A0A0R4IZX1

A2RS23

O60701

A2RSB1

Q3UER8

P08752

P16125

Q3TYD4

Q61553

Q8BWS5

Q8HWB2

E9Q6R7

E9Q0F0

Q9JJ28

A0A0B4J1E7

Q8K1I7

Q9CQI3

E9Q4Z2

G3X995

Q542T1

Q9DCW4

Q62093

Q9D8S5

Q9UN70

P62996

Q9CZ28

Q8R429

O55201

P54775

Q9Z2D8

P60174

Q80X68

Q9DCA5

P63330

E9PWQ3

Q8VDQ1

P43404

P24547

Q8BML9

Q61024

Q9WVM3

A0A0R4J2D8

Q8C0C7

P54103

Q64261

Q9R0Q7

P30613

P00326

Q9Z1K5

Q8BTZ7

Q8BH64

Q9UNW1

O95336

P28667

G3UWS4

H3BJW3

Q921K2

Q9CQW9

B2RWH3

Q13332

Q921M3

Q922D8

Q8C166

P62717

P08030

Q60749

Q8JZX5

D3Z780

Q3U431

Q5FWB7

Q3TY95

Q5BL18

Q05895

Q16610

Q9WV91

Q99497

Q542P8

P14131

P97801

Q8CI94

P37802

Q9CQQ8

Q12907

P61290

Q58E35

A0A0N4SVP8

Q9WTL7

P39876

Q545K4

Q58EW0

Q924K8

Q9WUK4

Q91YW3

A0A0R4J117

Q9Z2X1

Q9R1P1

Q8QZY1

P26039

H9H9T1

Q9WUA2

Q3U4X8

G5E8X5

Q54AJ5

Q9JKR6

Q9DAA6

Q61686

Q9R1P4

B0YIW2

Q9D1A2

Q8QZT1

P59325

H3BJQ7

D3YUP1

Q3TT94

P04114

Q62422

P46940

P11835

Q9D554

Q9DCT8

P12109

E9Q0K6

Q93099

A2AH85

P62858

Q3U2W2

P20152

Q810U5

P17426

P28828

O54984

Q790I0

Q3U5V8

Q99K48

Q99M46

Q3TZW9

Q8R550-2

Q4FK49

A2AF47

Q5M9L0

Q4KL81

Q9JM52-3

E9QPU1

Q9D3L3

Q921E6-2

P11352

P51680

Q9CU62

P10639

Q9CZ52

D6W5L6

P45376

Q9EQR5

P63001

Q5DQJ3

Q9WTR1

F8VPK0

E9Q7X6

O08992

P07954

Q9EQU5

P13707

E9QM75

F7DEU6

Q3UZG4

Q8BP48

Q5EG47

Q9ET22

G3X928

E9Q718

Q8VDM4

B1AHL2

E9Q912

Q62137-2

Q6ZWZ6

Q9ERU9

G5E829

P14543

P35293

O55234

P61202-2

Q91WM3

P02649

Q80X41

Q8R0J7

P97825

P18760

E9QNY6

Q9Y6R7

Q7TN58-2

Q9JM14

F8WHL2

A0A0J9YUL3

Q3UKJ7

P36536

A0A0G2JEA5

Q8BTS0

Q5M9M5

V9GX38

P70698

Q9D0E1

G3XAI2

Q9QXK3

P70168

Q61881

Q9CW46

D3Z627

E9Q035

Q3TIR3

P19324

Q3T9Z2

P13864

P60766-1

Q8C2K5-2

P84103

Q9QXK9

Q8VDD5

Q9QXY6

P14685

Q9Z127

Q9H7Y0

P54276

P48506

A0A0A6YX26

Q99715

Q12884

Q8R2Q8

Q9QZE5

P47963

Q3UB06

Q14520

Q9D9Z5

Q3UKW2

F5H5R8

P70423

Q8C669

Q9JIK5

Q58A65

Q8BKC5

Q80VZ7

Q61003

Q3TXS7

Q9Z123

P49182

P13020

P18052

Q3UFK8

O08804

Q9Z1R3

Q61206

A0A0U1RPL0

P20742

P06737

O88693

A2AVA0

G3XAK1

Q546G4

Q810D6

Q8R059

P18242

P09211

A0A0A6YVU8

P21836

A2RSY7

B7ZCF1

Q8C7V3

G5E8N5

P99026

Q60770

Q9CQ88

P56537

P68372

P17050

P62827

Q3UP78

O54962

Q99M28

Q9DBC7

P84084

Q8BYC6

Q6A0D4

A2AH25

Q5YLW3

P35279

Q6PHQ9

P50552

P07743

P63038

Q60972

B1AQP7

P14094

Q5SUA5

Q9NRV9

P80313

P14618

P99024

P40240

Q14019

Q549Q4

Q9QYJ0

Q8C266

G3X9X7

Q8BY89-2

P50395

G3UZK1

P68373

P46460

O08710

Q9Y3B4

Q9JMH6

Q66JS6

Q3TDA7

A0A0R4J093

Q91XV3

Q61171

Q9DBE9

Q6P9P6

Q9JHU9

Q8BQM4

P07738

Q9CZ04-2

E9QAQ5

Q9WVK4

Q9UNK4

P11087

Q497E4

Q8QZY6

Q8BHL7

A8DUK4

Q9CRT8

A0MNP4

Q8C708

P01591

P50454

Q9EPU4

O95445

Q8K224

Q9D868

J3KMX3

P21279

Q8CDN6

Q09143

Q64735

Q6YHK3

P97355

O35350

P18181

Q8BFQ4

Q2TBA3

P20334

F2Z4A3

O55029

Q9JKX6

Q9D0W5

Q9DCE9

Q3TEF1

O75083

Q8BV49

O60888

Q61152

A2AMU9

Q5SUZ8

Q8BIK4-2

Q5SSI6

Q99KQ4

Q6NZJ6

Q92954

Q91V55

Q9CSH3

Q62470-2

Q9JJ00

P07203

A0A0R4J0T5

P24668

P35951

Q9CZ30

Q9CQT1

Q6ZQ38

Q5M9P1

B2RUP2

Q6P069

Q769J6

Q3V117

Q9JL26

B4E1Z4

Q8R1Q8

Q8C7R4

O00391

A0A0A0MQC3

Q9CQ01

Q8QZR4

E9Q6E5

Q99KD5

Q8CAY6

O08917

Q9QUR6

Q9Z1Z0

Q3UH60

Q3U9E2

Q9H227

Q99L47

Q8R5C5

P62983

Q08943-2

O70400

Q96JK9

Q543N3

Q60737

Q9D753

Q9D8N0

Q9DBA8

P62071

Q9CYI4

P17301

P08228

Q3UZR8

O88983

Q542J3

P97300

Q01730

Q9JIM1

Q3UBY5

Q8JZZ5

B1ASP2

P97384

O95967

P04792

Q61074

P02301

P97452

A2A841

O35671

Q5Y5T1

Q92520

Q9CX00

Q6P9J9

Q9D8W5

Q80T21

O88587

Q564E8

Q3TTA7

P13597

Q8CG16

E9PXU2

Q9CXK8

Q9CPN9

E9PY39

J3QK52

Q6ZQ08-4

Q78PY7

E9QP62

P50580

P40189

Q3UDE2

Q8BG67-2

Q8K4Z5

P23298

Q3U0E8

Q9CQ80

B1AUN2

Q5XJF6

P06132

E9Q156

Q5SWN2

B1AT82

A0A0N4SW28

A2AEX6

P14211

Q3U7R1

P11031

Q8K1B8

Q8BJU0

O08553

P68510

P23381

A1L3C9

Q3UN51

P26041

Q07076

P70315

E9Q8I0

Q6P4T2

P23116

O35643

Q01853

Q91VH2

Q505D9

P04004

Q6P5F9

Q9CX56

Q99KK7

P31946

A0A087WQS2

Q9DBG5

Q9R0P5

P35700

A0A0R4IZZ0

Q9CQM9

P63271

Q9EPU0

Q9R1T4

Q61768

Q8VDN2

Q61699

Q8WWZ8

Q61321

Q8JZK9

P48723

Q52L97

P08556

P41241

P35908

P00742

B5B2N2

P62320

Q8C863

Q8BXL7

Q00651

G3V180

P70290

Q92820

Q9Z1D1

Q9D8B3

Q80SW1

B1ASL3

A2CEK3

Q9D3G9

P08567

P21399

Q9P265

Q8VE88

Q16531

P42227

E9QP00

P49290

Q9R0E1

P09972

A2AQ53

Q5SQ20

Q921M7

Q9HBI1

D3Z5I1

Q5NBZ3

A0A0B4J1H0

Q80X50-5

Q640N3

Q58EV5

D0QMC3

Q8VH51

Q99LU0

E9QAS8

P17751

Q9WU28

E9PUW7

Q5BLK2

Q9D6F9

P21981

Q9UGM3

Q9R061

P49888

Q15582

A0A140T930

P00966

Q8BVF2

Q6PDS3-3

A0A0B4J231

Q543H0

Q8VDP4

Q64449

O75874

O88307

A0A0A6YWM8

P32037

Q9JLB0

P48147

P62774

P62315

P18572

Q5M9K7

A2AGT5

A0A0R4J190

P28072

D3YUV1

Q62165

P47199

Q8NBM8

Q8BQ30-2

P50516

Q3TRJ1

A0A0A0MS08

Q9D0M0

F8WIT2

Q99JX3

P22646

Q91VB8

A0A0G2JIW1

Q3U367

B2RQQ8

Q99536

Q3V3E1

Q3TK95

D3Z7B5

Q9CQF9

P97371

Q8R146

Q9Z1A1

Q8BHL3

P47962

P23611

Q9CZ69

Q5HZI6

Q505L1

P47753

Q9CR16

Q78ZJ8

O15230

Q99PV0

O88653

Q9ES00

Q9Z0L8

Q03350

Q9JLV5

O70311

P62075

Q6V595

G3UXL2

E9QPI5

Q01581

D3YXG0

Q9Y617

Q01705

Q8K1I3

Q8K4G1

Q9QZD9

Q9JJY4

Q6ZWQ6

P47856

P59999

P31938

P61963

P98160

F5H2F4

O09173

Q9DCH4

Q9JHI7

A0A0G2JE99

P25786

Q7M6Y3

P16331

P60122

Q6P542

Q8R3L5

B2RTL6

Q8K157

Q9DCG9

Q569Z5

Q921D4

Q9D1M0

Q9R1P0

Q9WVA4

Q8JZQ9

Q4KML7

A2AGH5

Q8BVE3

Q8BY71

Q8VE70

Q0PD67

Q8K368

Q61029

Q78HU3

Q60668

Q6Q477

Q80XQ2

Q5T5C7

Q99J09

Q99MR6

A0A0N4SUH4

Q9Y6Z7

Q86UX7

Q6PR54-3

Q3UE92

G3UWD7

E9QMN5

Q3TJC2

Q99LF4

P29387

Q8VDQ8

Q9JII6

P08133

Q9CQR2

Q9CVB6

O35127

P49710

P48740

Q64737

P24161

P15261

Q61735-2

Q91X83

P10833

G3UW94

P29341

P35330

P10605

P14625

Q61599

Q3U9X2

Q8BG32

P01031

O55013

Q8VC30

Q5DW69

Q9ET01

Q61730-3

P01580

A0A140TA33

Q99LD9

A0A075B5J4

P61211

P02462

Q4VA10

Q8N0Y7

Q61090

O35857

Q9JKF1

A0A0R4J140

Q8BL97

O60462

Q5SQB7

Q99J77

P56399

Q06806

Q8R3N6

Q4FJL0

Q9ERN0

P20029

P49722

Q8BU03

P70677

Q9DCL9

B2RXS4

B2RRI2

P43274

P70670

G3X987

Q8BMA6

Q3TS38

Q3UQ44

P58546

P63104

Q3TN07

P12970

Q7TMY4

P13745

P01901

P04075

Q6S5J6

Q99LC5

Q9CSU0

Q7TML3

Q80Y52

Q62318

Q3UMF0

Q08093

P52480

Q3UAI3

Q99M31-2

P19823

O89103

P70349

Q14914

B2RVP5

E9QB02

P80511

P05106

Q922B2

Q91VK1

P61759

Q52KC3

P69905

Q62443

Q01320

Q9R0Q6

P09103

Q06138

Q9Z0P5

Q922R8

Q5SPX8

Q61235

O96009

Q9Z1N5

Q6P5B0

E9Q1G8

Q8BMN7

Q8C2Q3

Q545P4

Q8VIJ6

Q3U0V1

Q3U5F4

P11276

Q9Z0F4

Q61210-5

P23492

P62869

A0A0R4J049

Q9CPY7

P63028

Q8BG13

O88342

Q9Z103

Q3UBU9

F8VQC9

Q544K9

D3YYT1

P37804

P01831

F5BFH0

O54890

Q61753

O35103

Q3TEA8

Q3UQ28

P57784

Q5U4D8

Q5SUF2-2

Q9CPU0

Q91Z67

A0A0R4J0N8

P18206

E9Q3T0

E9QJS7

Q7TMM9

Q9CQM5

P14206

P07900

P19320

Q5M9P7

P08670

Q91YR7

Q9CQE8

P58252

Q9R1T2

Q8CCF0

Q61635

P00493

P55058

Q9JHU4

Q9DCC4

Q9JM76

Q91XL3

P48774

Q9CXS4

Q99JX7

Q64012

P16301

P12110

B1ATB3

Q8C129

Q6PFA2

Q3UPL0

P22752

Q6PE01

E9QAZ2

Q9DBG3-2

Q5FW97

O14974

F8WJA1

Q9QXD6

A0A0G2JFH2

Q8CFI7

Q9JIX8

P49257

Q9CRA5

Q8BGW0

P25206

Q8CFU0

D3Z7R7

O88569

O35206

P32067

P26043

Q61990

Q4VAE6

P09619

Q99LD8

Q569Z6

Q6P5D8

A0A0R4J2B2

Q9CRB2

P55288

P49962

Q4VAA2

A0A0R4J0R1

Q64281

P01869

P54116

Q61001

Q6ZWY3

O88543

Q8BGB5

Q8BRF7

P28658

P14148

B7Z9C2

P48036

P07996

G5E8J0

B2RUC7

Q91YI0

Q9D6L8

Q8VCC9

E9Q9M1

P07310

Q8VCG1

D6RFS9

P09411

Q6P5F7

Q3THS6

Q9DCY1

Q91W53

Q8WW22

Q9D1R9

E9Q4S7

Q8VBW8

Q62376

F8VPU2

P21956

Q9CZD3

Q9ERK4

P50502

Q62077

Q91YQ5

Q3UFY7

Q3UHZ7

Q3TE40

Q99JI6

Q3UIG0

Q5I0T8

P11047

P55263

Q3TQ70

P97823

P35550

P61161

Q8BT07

Q9ULV4

Q05D44

F6YVP7

E9PVA8

Q61739

B1AX58

P20908

B2RUR3

Q91VE6

P09405

Q6PGC1

Q3TGX0

Q9R182

P45591

P47757-4

Q9WUU7

E9PZ16

P70441

Q9CY46

P12814

O00533

Q64337

Q62048

Q9Z0K8

P46061

Q8C788

P61979-2

P07742

Q3U9G9

Q9QZC2

P60843

P29391

Q07797

Q4VBG1

P62281

Q8BHN3-2

P62137

Q5SUH6

Q3UW53

P28653

A2ADY9

Q9P2F6

Q8R010

P28867-2

Q9D2V7

Q99MK8

Q9WUM4

P29401

Q8BK67

Q9CQN1

P27661

P0CW03

Q8C405

Q91YH6

Q9JI48

A0A0R4J1E2

O35714

P56480

Q8BVK9

Q80VA0

P27773

Q9QUM4

E9Q3G8

P58281-2

Q9JIF0

O54901

E0CYV0

Q62384

G3X9T7

O08788

E9QKR0

O88456

P48678

Q8R050

O15067

O70423

Q9ESU7

A0A0R4J1Q7

P02753

G3X8Y3

B1AYC9

P41155

P00740

O35598

P62334

Q62351

P97855

Q6DFW5

A0A0B4J1R4

P14152

P12955

Q9QUM0

Q544H9

Q01813

Q9Z204

Q8CIN4

Q8BIQ5

Q3UDC3

Q3TYX3

Q9QZQ8

Q8CI32

Q8C6G8

Q9Z2L7

Q9JIF7

Q8CBC8

F8VQL0

P41245

D3YYL7

E9QLL8

Q8R1B4

Q8BK64

Q9EST5-2

Q9DBJ1

Q58EV4

P01023

Q61982

Q80UL9

Q3UTP8

F8WGM5

Q9QXD8

P11499

O08547

P08882

Q62418

O55098

Q9CPW4

Q3ULT2

Q9CPV4

Q9DC51

Q8VBV7

P34960

Q6ZWX6

Q9WUD1

P68369

Q8BH24

K3W4S6

P19788

Q4V9X1

Q9CYN9

Q16473

O08532

Q61510

Q64511

Q02248

E9QNL5

Q9DB34

Q59IW6

P16546

A2APM2

Q8K1X4

Q3U1S6

P12815

P39428

A2AUD5

G3UW85

Q3V014

Q542X7

Q3V1P4

Q9JJI8

Q9D1C8

Q9QUM9

P43276

B1ATD2

P68104

Q00612

Q3U9N9

Q9D0T1

P30086

P61923

O35969-2

Q6EMK4

Q9D7M1

Q99N09

P60766

Q91VW3

Q7LGC8

Q9D7S9

Q80U72-3

Q60675

P00558

Q8R555

Q8VBV3

Q62465

Q9Z1Q5

P50518

P19973

P80315

P31041

P26038

Q04519

P28474

P58044

B1AU75

Q9D898

P01849

F8VQC1

Q99LX0

Q9JHJ0

H3BK50

Q6A0A9

Q9JLI0

Q8VDM6

P49429

Q9D8C2

Q6ZWU9

Q61990-2

E9PV24

P70232

Q9CQ79

Q4FJW7

Q99LB6

Q8C483

Q5HZY7

Q3UNK5

P09055

Q3UXS0

Q8K0T7

Q63932

E9Q414

G5E8V9

Q8BZM1

Q8R4V5

O00299

P30416

Q60953

E9PXY8

Q8BGK6

H9KV04

Q3TF14

Q91Z40

P63024

Q924C1

P50990

P13609

Q7TSG5-2

O35654

P49189

P35762

Q3UPH1

Q13093

Q9D0B6

Q9JK23

P98064-2

P19096

E9PWR4

Q3UDP9

P08071

P84078

A0A0G2JEC4

Q545V8

Q8VDJ3

P05386

Q3TJZ6

P04264

P01731

Q8K2Q7

P62849

Q9CQ10

Q5SUR0

A2AD85

P09470

A0A0R4J008

Q9JHK4

P52430

Q8C8K1

O35490

Q8BGD9

Q02105

P15535

Q9EPK2-2

Q542F4

F6VW30

J3QNK8

Q99PT1

Q9DB60

P97370

P26883

Q9CR00

Q60631

Q8CF98

O08528

Q7TT37

Q9JJU8

Q9JIZ9

A2AI08

Q3UBS3

P18428

Q61790

P05201

P01024

Q8C570

P10768

P26369

Q04750

Q3UYV9

Q9Y240

P11440

P24821

Q8CFQ3

Q8CG76

P34884

Q3B7Z2

F6Q8A4

P63087-2

O75829

P35235

Q5SS83

P27808

O35226-2

O60763

Q61187

Q9CXF4

B2RXV4

Q14AI7

Q497E9

Q99J83

P62918

P21333

Q50HX4

Q8K2Y3

Q9D0R2

Q61543

E9PUU4

Q6ZWZ4

Q9D892

Q9Y490

P17809

A2A432

P63037

P18085

Q920Q8

P67871

O70318

P20618

E9Q3L2

P62301

A0A0U1RQQ9

Q14AF6

Q9CYA6

Q4VAG4

Q9CQW1

Q8R5H1

Q3TUQ5

Q00422

Q3TAQ9

B2RX66

P46664

P62305

Q059P4

P50543

P63213

F6Q8D3

A0A075B694

Q91Y97

Q91Z25

E9PYD5

Q8CJF8

Q76LV0

P29352

Q58E64

P63005

O88844

Q00PI9

P27612

Q9NRN5

P84091

Q9Z1M8

Q9R1Q7

Q3UKJ6

Q60864

F8WHU5

P24043

P97798

E9Q7G0

P35979

Q8BT60

Q6ZWZ7

Q059T9

Q9CY66

Q8C2Q7

Q3THE2

Q4FJX4

P43275

Q99KN1

P62841

P31266

O70194

Q9Y6M4

O00187

Q80V42

Q8CHR6

E9PZC3

F6ZFU0

Q5XJY5

Q9QYG0-2

F8VPL5

Q8CFZ4

Q5MJS3

Q9Z2U1

Q9Y696

Q8K274

Q91XD6

P61164

P30412

Q80X90

Q9H0W9

P62830

Q64374

Q0PD65

Q6P6L6

Q8R317

Q8C0D5

Q5SVP3

Q9DBR7

Q62189

P23921

P11157

Q9CYG7

A0A0G2JGL0

E9PK25

P19157

Q8BH43

A2A7S7

P47811

Q9JHK5

Q61166

Q15113

Q3UXZ9

Q561M1

Q3U0D7

P31324

P20934

Q8K352

Q61233

Q9DBG6

Q3UII2

Q8BGQ7

Q99KH8

Q9BX97

P57716

P14115

Q69ZR2

Q8R2P8

P98063

Q5EBQ2

F8WJD4

Q8R379

P17918

Q61033

P53634

Q9CQR6

Q99LT0

O89079

Q9D1P4

Q6PAR5-4

Q5SXR6

Q91VR5

P97333

P83940

P29351-2

Q9D2M8

Q9D1G1

Q9DB27-2

Q9CQ22

Q96M27

Q6P5F6

Q80SZ7

H7BZJ3

E9QN70

O35250

Q6GT24

Q99KP6-2

P97822

P62080

Q6ZWV3

Q9R118

Q91VI7

Q9EPL8

Q8R3X6

O35134

B2RQC6

P12111

Q9QXK7

P02452

Q62261

P97310

Q8VC77

P06745

O35452

Q60841

Q9QZ06

G5E866

Q3U2G2

P35443

Q3UN35

F6TLX2

Q5BLK1

Q9QZ88-2

Q64378

Q64514-2

D3Z312

Q05816

P82198

P28481

Q9Z0E6

**Supplementary Table 2.** D1E differential proteins. This list includes proteins either only detected in D1E, or, at least 1.5-fold higher in abundance in D1E than in D3E, as calculated by a comparison of the average abundance of each detected protein in reference to GAPDH, the internal control.

| **ID** |
|---|
| P01031 |
| Q92520 |
| Q99KQ4 |
| Q8CAY6 |
| Q3V117 |
| Q497E4 |
| P28474 |
| Q9JII6 |
| P45376 |
| Q9JLI0 |
| P24549 |
| Q8R0Y6 |
| Q3U367 |
| P04075 |
| Q91Y97 |
| P09972 |
| Q9HDC9 |
| P84078 |
| Q61024 |
| P00966 |
| P31939 |
| P15535 |
| Q8BWP8 |
| O35490 |
| E9PZC3 |
| P24270 |
| P16152 |
| Q7LGC8 |
| G5E8X5 |
| P12960 |
| Q12860 |
| O88587 |
| Q99LD8 |
| Q3U741 |
| Q9JIK5 |
| P54823 |
| Q3UKJ6 |
| Q9ESX5 |
| O08553 |
| Q12805 |
| Q9D1Q6 |
| H3BKH6 |
| Q3V1P4 |
| Q93063 |
| P00488 |
| Q5MJS3 |
| P35550 |
| Q8K1B8 |
| P30416 |

P02751
P11276
Q8K157
O35969-2
Q9CZD3
Q9Z0E6
Q9CPU0
F6TLX2
Q3THK7
P05201
P13707
P22352
Q16772
P13745
P48774
P09211
G3V4T6
Q61035
O09173
Q20BD0
P49429
P00493
Q80Y52
A0A0G2JIW1
P20029
E9PZ16
P98160
O75874
P24547
Q8BMJ2
Q3U9G9
P04180
P16301
G5E8N5
P07195
P16125
P97823
Q9WTL7
Q91X83
Q3THS6
P14152
Q9CQ65
P35579
Q8VDD5
Q9QWR8
Q99J77
F5H5R8
Q6DFW4
Q7TQI3
Q921K2
Q9CQF9
P52209
A2CEK3
Q61753

Q9UNK4
P55058
Q3UFS5
P23492
P62937
P17742
Q9QZH3
H3BJQ7
Q6GT24
Q9Y617
Q543K5
Q922I7
Q9R0Q7
Q14914
Q8CI94
P06737
Q9ET01
P09417
O00391
Q78ZJ8
Q0PD67
Q05144
P62827
Q99JI6
Q9D0I9
Q64374
Q4VA10
P42208
Q543J5
P50431
Q00796
E9QNL5
P49888
Q9D0R2
P29401
P40142
Q64511
P60174
P17751
P61089
Q3TS38
Q9EPU0
P18206
P32921
P63104
Q9R182
Q9Y240
E9QLW5
G3XAK1
P20774
P54289
O00299
Q9QYB1
P55263

P55264
Q542X7
Q04447
A0A0R4J093
Q9R0N0
O08528
O55222
O60462
P12382
Q8C605
P00558
P09411
P14618
A0A0R4J097
Q06806
Q9CPN9
J3QNK8
P68134
Q4KL81
P97429
Q9P2F6
Q99PT1
Q91Z25
A6H6K1
O54962
P07743
E9PGA6
P07358
Q01518
P40124
P47753
Q5M9P7
Q6NS45
Q3U4U6
P80316
Q3U429
Q3UII2
E9PK25
O75829
Q99715
O35206
E9QPX1
P11087
P02452
P08123
P02462
P12109
P12110
P12111
E9PWQ3
G3X995
Q9WUM4
Q14019
Q9CQI6

Q62418
E9PV41
E9QM99
P63168
O95967
Q9QXY6
E9QK48
Q6V0I7
Q8CIB5
E9PV24
C9JC84
Q3UER8
Q059P4
Q8BTM8
P21333
Q06828
P50396
P50395
Q61598
E9QAZ2
F6YVP7
P02301
Q9QZQ8
Q9NRV9
P43275
P43276
Q149Z9
P43274
Q8CGP2-2
Q5EBP8
Q9Z2X1
G5E924
Q9D0E1
P14625
Q3UAD6
P48723
P04792
P14602
P35330
K4DIA0
Q61635
A0A0R4J0S2
P01751
A0A0G2JE99
A0A0B4J231
Q80UL9
P01591
P04264
P35908
P35527
P24043
E9QN70
F8VQJ3
Q61233

E9QP62
P14733
Q8R001
G3X9Q0
P19788
E9Q3X0
B7Z9C2
E9PW66
P13595
Q8K1X4
Q9QYG0-2
P14543
P10493
E9QN31
Q9D6Z1
E9Q7G0
Q8QZR4
Q8WWZ8
Q9NRN5
O35103
Q9HBI1
Q9NPG4
Q9UN70
Q15113
P30086
Q5EBQ2
P08567
Q9DC11
Q62009
Q92954
Q99PV0
B1AT82
Q3TXS7
Q8VDM4
Q61838
P02753
P26043
Q9HD89
Q91VI7
P47962
Q5EBQ6
P62281
Q5M9L7
Q01730
P80511
Q3TGX0
A0A0U1RQQ9
E9Q1G8
E9QLL8
P50454
P19324
Q921M3
Q8VIJ6
D6W5L6

A0A0R4J2D8
Q62376
Q8K1I3
P56873
P50502
P54116
B2RUC7
A2AVA0
P37802
Q9WVA4
O08710
Q15582
P07996
P35443
B2RTL6
P39876
Q9Y490
P26039
P24821
A0A0B4J2C3
P01849
P68369
P68373
A0A0A0MQA5
Q9D6F9
P68372
Q8K0T7
Q6EMK4
P50552
P70460
P20152
E9QPU1
O75083
O88342
Q6ZQL4
Q5SS40
A8IP69
F6VW30
P09470
Q76LX8
Q769J6
Q8CG16
P01024
A0A0G2JPR0
P07384
K7ERG9
Q9D1A2
Q9Y6Z7
P53634
Q9WUU7
P00742
P00740
Q9Z0L8
P08882

Q14520
P08071
P98064-2
P48740
O00187
P14780
P30101
P27773
A2RS23
Q9QUR6
Q792Z1
I3L0U2
Q9R1P4
P49722
Q58EV4
Q9R1P0
Q9QUM9
O14818
Q9R1P1
P99026
Q60692
Q60841
Q9QXD6
P70695
Q8N0Y7
P62141
P63330
Q64487-10
A2A8L5
Q13332
Q7TPR4
P14211
Q6ZQ38
P27661
Q8BV49
Q96JK9
Q921D4
Q9R190
P58546
Q3U2W2
P97798
Q61982
O70400
P62196
Q60973
P11031
Q62318
P62960
P31946
A0A087WQS2
Q9D8N0
Q3ULL5
Q8R1B4
Q9QZD9

Q6NZJ6
F5BFH0
Q9UGM3
P11717
Q07113
Q61730-3
P40189
Q9QUM0
P09055
P05106
A0A0R4J0I9
Q64449
Q9UBG0
O14786
P97333
Q6PDS3-3
P15702
P19320
P01023
Q546G4
P08758
E9Q414
P04114
B0YIW2
O95445
Q9Z1R3
F6Q8D3
O55143
Q9CYN9
Q9JIF7
O55029
Q16610
P21995
Q8BG05
Q8VEK3
Q9DB05
P61971
Q8CG48
Q8C788
A0A0R4J0R1

**Supplementary Table 3.** D3E differential proteins. This list includes proteins either only detected in D3E, or, at least 1.5-fold higher in abundance in D3E than in D1E, as calculated by a comparison of the average abundance of each detected protein in reference to GAPDH, the internal control.

| ID |
|---|
| P01580 |
| P34884 |
| P26883 |
| P32883-2 |
| Q9WTI7 |
| Q4FJL0 |
| P63321 |
| P97855 |
| P97310 |
| Q9JJ00 |
| Q9JIZ9 |
| P61226 |
| Q9CQI3 |
| P67871 |
| P11440 |
| P43404 |
| P70372 |
| Q5M9L0 |
| Q61699 |
| Q5M9M0 |
| Q5I0T8 |
| P41105 |
| P62889 |
| A0A0A6YX26 |
| P62911 |
| P62841 |
| Q9CZX8 |
| Q5BLK2 |
| P62852 |
| P62855 |
| A0A0R4J0T5 |
| Q9JI48 |
| Q3UEB3 |
| P35979 |
| Q4VAG4 |
| Q6ZWZ4 |
| Q58E35 |
| Q9D8S5 |
| P62320 |
| P63163 |
| Q99N09 |
| D3Z2H9 |

O54901
P18181
Q3TEK8
Q6PFA2
Q64735
P05533
Q01965
Q3TA56
P61290
Q91Y47
Q02105
P70670
O70251
Q6ZWX6
Q9DCH4
P62267
P22646
B1B507
Q9QUM4
Q9D1C8
Q52L97
P97370
P53986
Q3UDP9
Q09143
Q9Z127
P01590
Q3TZH4
Q3UIG0
P18572
Q5SQB7
P32037
P08030
P13597
Q9CYL5
P47963
P01887
P01831
Q6ZWV3
P63213
P10852-2
P14206
Q58EW0
P35762
P62918

Q549Q4
Q99KN1
H7BX95
Q58E49
Q8C708
A0A0R4J1E2
P12970
S4R1S4
Q542I2
Q564E8
P35700
Q01853
Q4FK49
Q543H0
P62835
E9QB02
P08249
Q3TQ70
P68040
Q922B2
P62071
Q99JI4
Q00612
P08752
Q9QUJ7
Q99KP6-2
Q3TQX5
Q9DCL9
B2RQC6
Q3V3E1
Q5YLW3
O08992
Q01320
Q9JIF0
Q3UL22
Q9DC51
P19157
P84084
Q8BGQ7
P17918
P11499
Q922D8
Q1HFZ0
Q8CGC7
Q790I0

Q8C266
F8WIT2
Q9Z1Q5
A0A0A6YWM8
Q99KH8
E9Q696
O55098
O70293-2
P25444
Q5M9M4
Q9CVB6
Q9JLZ6
Q3UKW2
Q8BP67
P62849
P80315
Q4KML7
Q9CR57
P61255
Q5SUA5
A2APM2
Q9Z123
Q92747
A0A0R4J117
Q8BL97
P34022
Q545X8
P70441
P80317
Q60864
P27659
P63037
Q91V55
P99027
Q6ZWZ7
P80313
Q5XJF6
P59999
Q8QZY1
Q5SXR6
Q9ES61
Q9D1R9
Q497E9
Q9WU78
Q99JX4

P62830
Q3UN51
Q3U2G2
P97351
P14115
Q8R0J7
P21956
Q9JM76
Q5M8R8
P11983
E9PYL9
P18760
P07356
P01899
P12815
Q54AJ5
P26041
P14131
P97371
P01901
Q9WVK4
O09167
P61161
Q92820
Q64514-2
Q8BVQ9
P70677
P28843
Q61187
P14148
Q9WTM5
Q921F2
P50580
Q9Z0N1
Q6ZWY3
Q8R050
P62264
P29341
Q61735-2
D3Z627
Q3UP78
P31041
A2AW86
Q61790
O35714

P11835
Q61003
Q62351
Q9ESU7
Q91YH6
P17809
Q9QZE5
Q8VDN2
Q3U0D7
P61923
Q8BY89-2
